# An important role of PHRF1 in dendritic architecture and memory formation by modulating TGF-β signaling

**DOI:** 10.1038/s41598-020-67675-2

**Published:** 2020-07-02

**Authors:** Ting-Wei Shih, Li-Jen Lee, Ho-Ching Chang, Hung-Wei Lin, Mau-Sun Chang

**Affiliations:** 1 Institute of Biochemical Sciences, College of Life Science, National Taiwan University, No. 1, Sec. 4, Roosevelt Road, Taipei, 10617 Taiwan; 2 Graduate Institute of Anatomy and Cell Biology, National Taiwan University, Taipei, Taiwan; 3 Institute of Brain and Mind Sciences, National Taiwan University, Taipei, Taiwan; 4 Neurobiology and Cognitive Science Center, National Taiwan University, Taipei, Taiwan; 5 Institute of Biological Chemistry, Academia Sinica, Taipei, Taiwan

**Keywords:** Neuroscience, Learning and memory, Hippocampus

## Abstract

PHRF1 is involved in transforming growth factor β (TGF-β) signaling to constrain the formation of acute promyelocytic leukemia (APL) in mouse APL models. PHRF1 also participates in modulating non-homologous end-joining. However, the role of PHRF1 in mammalian dendrite architecture and synaptic plasticity is unclear. Here, we investigated the role of PHRF1 in dendritic formation in the murine hippocampus using Camk2a promoter driven-iCre recombinase to conduct a PHRF1 conditional knockout, namely PHRF1^Δ/Δ^, in the forebrain region. PHRF1^Δ/Δ^ mice developed normally, but exhibited anxiety-like behaviors and displayed defective spatial memory. Alterations of dendritic complexity in apical and basal dendrites of pyramidal neurons were noticed in PHRF1^Δ/Δ^ mutants. Furthermore, electrical stimulation in the hippocampal CA1 region after the TGF-β1 treatment showed a reduced synaptic plasticity in PHRF1^Δ/Δ^ mice. Immunoblotting analysis indicated that PHRF1 ablation affected the TGF-β signaling. Collectively, our results demonstrate that PHRF1 is important for the dendritic architecture and required for spatial memory formation in the hippocampus.

## Introduction

Pyramidal neurons are mainly associated with cognitive functions in most mammalian forebrain areas, including the cerebral cortex, the hippocampus and the amygdala^1^. The hippocampal circuits exhibit the well known ‘tri-synaptic loop’. The entorhinal cortex connects the hippocampus with its projections to the dentate gyrus (DG) region. The DG projects to the CA3 region via the mossy fibers. CA3 links to the CA1 region via the Schaffer Collateral pathway. Finally, CA1 circuits back to the entorhinal cortex, completing the tri-synaptic loop^2^. In addition to the tri-synaptic loop, the critical functions of pyramidal neurons in the hippocampal CA1 region are the way they respond to surrounding stimuli and produce excitatory outputs to its postsynaptic targets. This process of information integration is largely dependent on the helm of dendritic structure and function^3^. The dendritic tree of a pyramidal neuron has two distinct domains: the basal and the apical dendrites, which are derived from the soma of pyramidal neurons. All pyramidal neurons have several short basal dendrites, and one large apical dendrite that can connect the soma to the tuft of dendrites. This main apical dendrite bifurcates and gives rise to the tuft at a variable distance from the soma^3,4^. An understanding of the dendritic complexity is required to elucidate their sophisticated functions in receiving inputs, and transforming these signals into cognitive learning and spatial memory. In addition to cognitive memory and spatial navigation, anxiety disorders are found to be associated with the hippocampus via its functional dorsoventral axis. The dorsal hippocampus contributes to cognitive learning and memory. By contrast, the ventral hippocampus modulates emotional regulation^5,6^. It is the dysfunctional ventral, not the dorsal, hippocampus that exhibits anxiety related behaviors, with minimal effect on spatial learning^7,8^, whereas dorsal hippocampal lesions affect spatial learning without affecting anxiety-related measures. Furthermore, ventral CA1 (vCA1) is enriched in anxiety cells that are activated by anxiogenic environments and are required for avoidance behaviour^9^.

PHRF1 is an E3 ligase, which is responsible of the ubiquitination and degradation of target proteins. One prospect of the PHRF1′s functions is involved in the regulation of acute promyelocytic leukemia (APL). PHRF1 mediates the degradation of the TG interacting factor (TGIF) to promote the function of the cytoplasmic variant of the promyelocytic leukemia protein (cPML) in TGF-β signalling^10^. The binding of PHRF1 with the TGIF is interfered by oncogenic PML-RARα fusion protein. Consequently, the TGIF is not degraded, and the cPML is inactivated, thereby inhibiting TGF-β signaling. By contrast, PHRF1 overexpression restores TGF-β cytostatic signaling and suppresses acute promyelocytic leukemia (APL) formation in mouse APL models^11^. Recently, Wang et al*.* described that PHRF1 may attenuate the proliferation and tumorigenicity of non-small cell lung cancer cells. Overexpression of PHRF1 arrested the cell cycle in the G1 phase and inhibited H1299 cell proliferation, colony formation in vitro*,* and growth of tumor xenograft in vivo^12^. Additionally, we report an alternative function of PHRF1 in modulating non-homologous end-joining (NHEJ). PHRF1 combines with dimethylated and trimethylated H3K36 and NBS1 to promote NHEJ and stabilizes genomic integrity upon DNA damage insults^13^.

Although many factors have been found to participate in learning and memory in the hippocampal circuits, the role of PHRF1 in the hippocampal dendritic architecture and synaptic plasticity remains unclear. Here, using a Camk2a-iCre mediated forebrain-specific deletion strategy, we aimed to delineate the functional phenotypes of PHRF1^Δ/Δ^ mice by means of anatomical, elctrophysiological and behavioral examinations. In PHRF1^Δ/Δ^ mice, the complexity of dendritic architectures in the hippocampal neurons was altered, while hippocampus-mediated spatial learning and memory was impaired. Unexpectedly, PHRF mutant mice displayed anxiety-like behaviors. We measured the electrophysiological recordings in the hippocampal CA1 region post TGF-β1 treatment. These results lead us to conclude that PHRF1 is important for the formation of hippocampal dendritic structure and memory formation.

## Results

### Generation of forebrain-specific PHRF1 knockout (PHRF1^Δ/Δ^) mice

**Figure 1:**
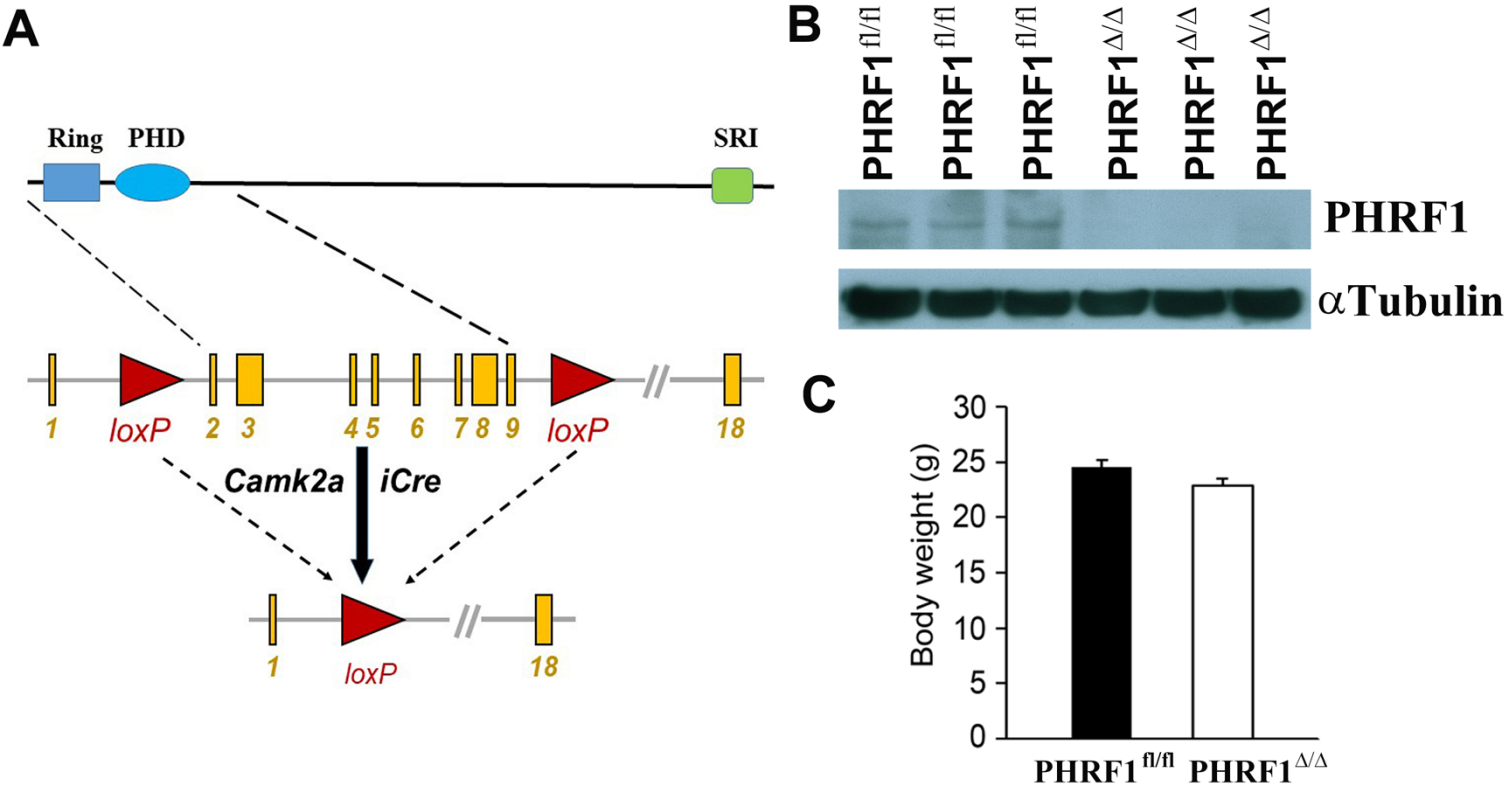
Conditional disruption of PHRF1 gene in mice. (**a**) Schematic representation of the mouse *PHRF1* gene flanked by two loxP sites between exon 1 and exon 10 of *PHRF1* gene. (**b**) Immunoblot analysis of hippocampal extracts prepared from PHRF1^fl/fl^ and PHRF1^Δ/Δ^ littermates. α-Tubulin was used as a loading control. All Western blots were processed in identical conditions and cropped from Supplementary Fig. S4a. (**c**) Comparison of body weight from PHRF1^fl/fl^ and PHRF1^Δ/Δ^ littermates (n = 5).

To assess the impact of PHRF1 ablation on brain neurons, a conditional knockout approach was used. A calcium/calmodulin-dependent protein kinase II alpha (*Camk2a*) promoter is known to drive the Cre recombinase expression in the forebrain excitatory neurons, such as cortical projection neurons and hippocampal CA1 pyramidal neurons^14^. We first introduced two loxp elements to flank exon 2 and 9 (a.a. 1–343) of the murine *PHRF1* gene by using the Crispr/Cas9 system. Mice carrying flanked PHRF1 (PHRF1^fl/fl^) were then crossed with Camk2a-iCre transgenic mice to produce forebrain-specific PHRF1 knockout (PHRF1^Δ/Δ^) mice. In this design, the E3 Ring domain and PHD domain (a.a. 109–153 and a.a. 188–232, respectively) were deleted in the presence of iCre recombinase (Fig. 1a). Immunoblotting analysis confirmed a significant reduction of PHRF1 in the hippocampal extracts prepared from adult PHRF1^Δ/Δ^ mice, compared with PHRF1^fl/fl^ controls (Fig. 1b). PHRF1^Δ/Δ^ mice were viable with no noticeable apparent defects. The body weight of adult PHRF1^Δ/Δ^ was similar to that in control mice (Fig. 1c).

### Loss of PHRF1 in the forebrain produces anxiety-like behaviors in mice

**Figure 2:**
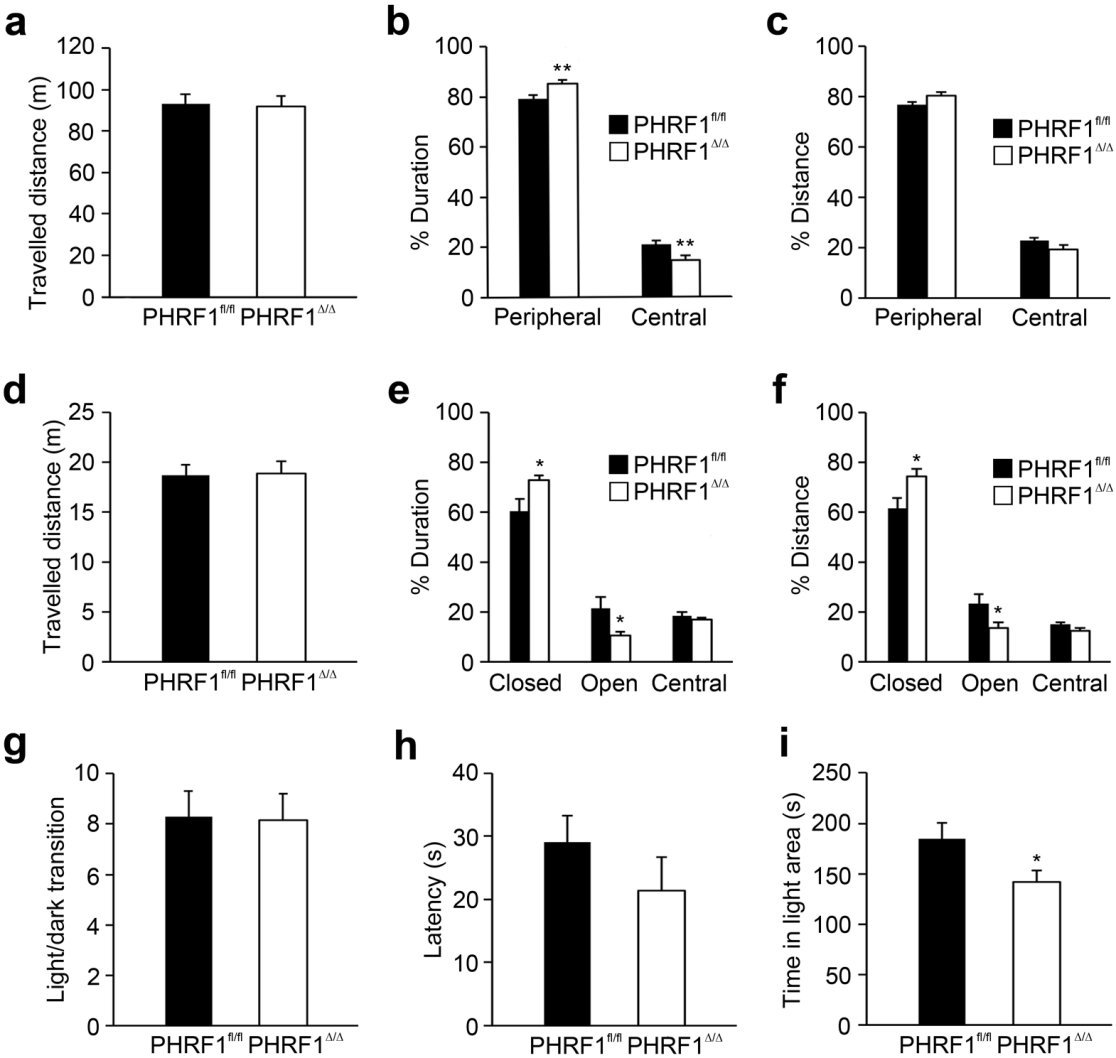
Behavioral tests. (**a**–**c**) Open field test. The total travelled distance and the percentage of time and distance spent in central and peripheral regions were recorded and quantified. (PHRF1^fl/fl^ n = 15, 12 males, 3 females; PHRF1^Δ/Δ^ n = 13, 10 males, 3 females) (**d**–**f**) Elevated-plus maze test. The travelled distance and the percentage of time and distance spent in closed arm, open arm and central zones were analyzed. (PHRF1^fl/fl^ n = 6, 4 males, 2 females; PHRF1^Δ/Δ^ n = 7, 3 males, 4 females) (**g**–**i**) Light/dark exploration. The time spent in the light region, transition times between the two compartments and the first entrance latency to the dark compartment were quantified. (PHRF1^fl/fl^ n = 7, 4 males, 3 females; PHRF1^Δ/Δ^ n = 7, 3 males, 4 females). Data are mean ± SEM. *p < 0.05, **p < 0.01, two-tailed unpaired Student t-test.

Since there was no apparent abnormality in PHRF1^Δ/Δ^ mice, we first examined their locomotor activity and emotional status using the open field test. An individual adult PHRF1^fl/fl^ or PHRF1^Δ/Δ^ mouse was placed in a novel open field arena and allowed free exploration. During the 30-min exploration period, the movement of mouse was recorded and analyzed. The total travel distance in the open field arena was comparable between PHRF1^fl/fl^ and PHRF1^Δ/Δ^ mice (Fig. 2a), indicating that locomotor activity is not affected by the removal of PHRF1 from the forebrain. However, PHRF1^Δ/Δ^ mice spent less time and traveled shorter distances in the central region compared with PHRF1^fl/fl^ mice (Fig. 2b,c), indicating a greater anxiety level in PHRF1^Δ/Δ^ mice. This notion was supported by the differential travelled distance in peripheral and central areas. PHRF1^Δ/Δ^ travelled longer distances in the peripheral area and shorter distances in the central region (Fig. 2c). The anxiety-like behaviors in mice was further examined using an elevated plus maze and the light/dark box test. On the elevated plus maze, the travelled distance was comparable between the two genotypes (Fig. 2d), again, indicating similar locomotor activity between the two groups. Notably, compared with PHRF1^fl/fl^ mice, PHRF1^Δ/Δ^ mice spent more time and travelled longer distances in the closed arms (Fig. 2e,f), exhibiting a sign of anxiety-like behavior. In the light/dark box test, the numbers of transitions between the two compartments were similar among the two groups (Fig. 2g), signifying comparable locomotor activities. However, PHRF1^Δ/Δ^ had a shorter latency to enter the dark compartment (Fig. 2h) and spent less time in the light area (Fig. 2i), compared with PHRF1^fl/fl^ mice, indicating a sign of anxiety in PHRF1^Δ/Δ^ mice, especially in unfamiliar or stressful environmental conditions. Male and female PHRF1^Δ/Δ^ mice exhibited similar anxiety like behaviors; however, some of gender behaviors were not statistically significant due to a small sample size (Supplementary Fig. S1).

### Forebrain PHRF1 ablation impairs learning and memory

**Figure 3:**
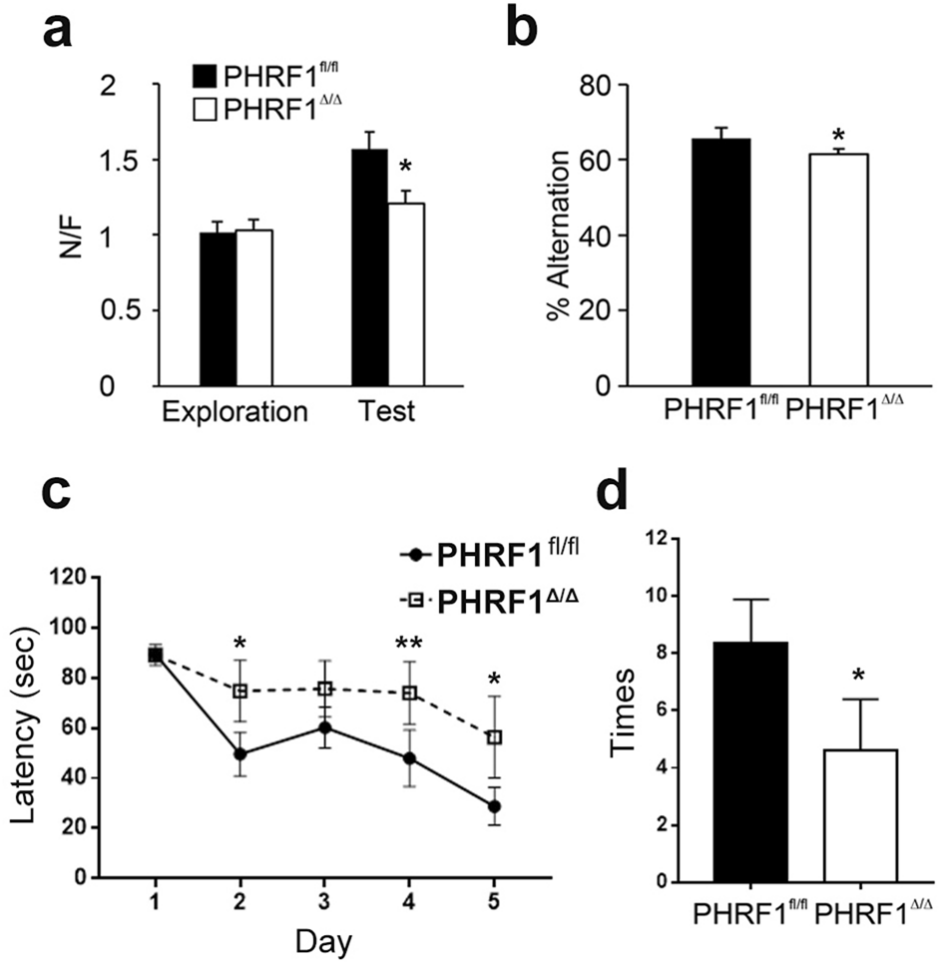
Learning and memory test in PHRF1^Δ/Δ^ mice. (**a**) Novel object recognition. In the test phase, the discrimination ratio of the familiar object and novel object were quantified (n = 5, all males). (**b**) Y-maze test. Each mouse was placed at the end of one arm and allowed to move freely through the maze during an 8-min session; the number of alternations were analyzed. Data are mean ± SEM. *p < 0.05, two-tailed unpaired Student t-test. (PHRF1^fl/fl^ n = 13, 11 males, 2 females; PHRF1^Δ/Δ^ n = 12, 9 males, 3 females) (**c**) An individual mouse in the PHRF1^fl/fl^ and PHRF1^Δ/Δ^ groups was placed in a water maze and allowed to swim to the hidden platform four times a day for five days. The swimming path and duration in quadrants of the water pool were recorded, and the latency to locate the platform was averaged and analyzed each day. *p < 0.05 by student’s t-test. (n = 12; 8 males, 4 females). (**d**) For the probe trial on Day 6, the hidden platform was removed and the number of times the mouse swam to the empty area in 90 s was recorded and analyzed (right panel). * *p* < 0.05 by student’s t-test. (n = 12; 8 males, 4 females).

**Figure 4:**
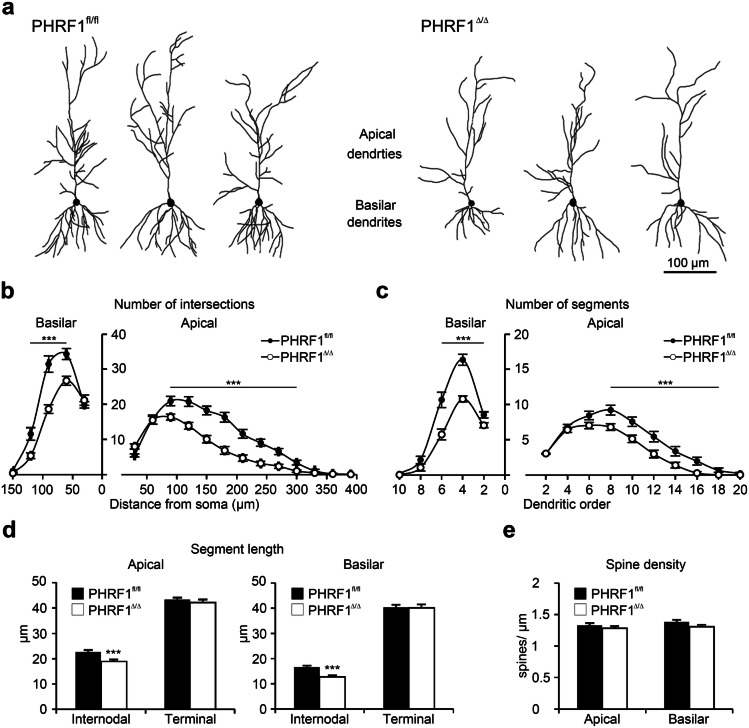
Dendritic architectures of CA1 pyramidal neurons. (**a**) Examples of reconstructed CA1 pyramidal neurons from both genotypes. (**b**, **c**) Dendritic complexity was assessed by Sholl analysis and the number of segments of each dendritic order. (**d**) The length of internodal and terminal dendritic segments. (**e**) Density of dendritic spines. n≧30 neurons from 7 mice for each genotype. Data are mean ± SEM. *p < 0.05, **p < 0.01, ***p < 0.001, two-tailed unpaired Student t-test.

**Figure 5:**
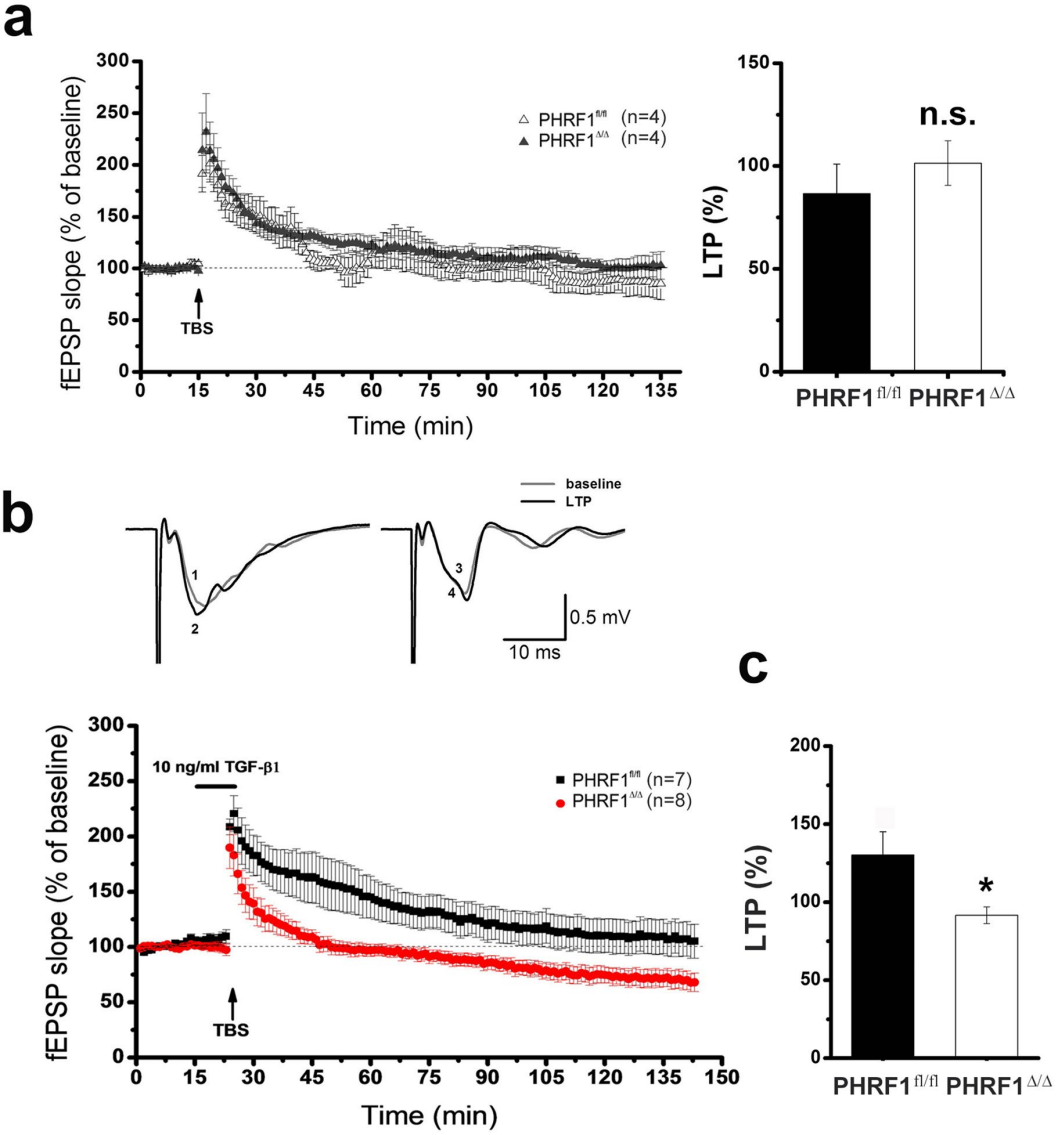
Theta burst stimulation (TBS) induced synaptic changes in the hippocampal slices. The extracellular field-EPSP (fEPSP) was recorded in hippocampal slices following the stimulation of the Schaffer collateral pathway. (**a**) An arrow indicates the start of theta burst stimulation (TBS). Note that there is no statistical difference (n.s.) between genotypes. (**b**) The hippocampal slices were incubated with TGF-β1 (10 ng/ml) for 15 min before the application of TBS (arrow). The black and red dots represent averaged points of fEPSP slope from PHRF1^fl/fl^ and PHRF1^Δ/Δ^ mice, respectively (n = 7 slices from 4 PHRF1^fl/fl^ mice; n = 8 slices from 4 PHRF1^Δ/Δ^ mice). Upper panel shows the representative tracings before (grey) and after (black) TBS of PHRF1^fl/fl^ and PHRF1^Δ/Δ^ mice. (**c**) At 60 min after TBS, the mean fEPSP values were analyzed by one-way ANOVA. Data are mean ± SEM. *p < 0.05.

**Figure 6:**
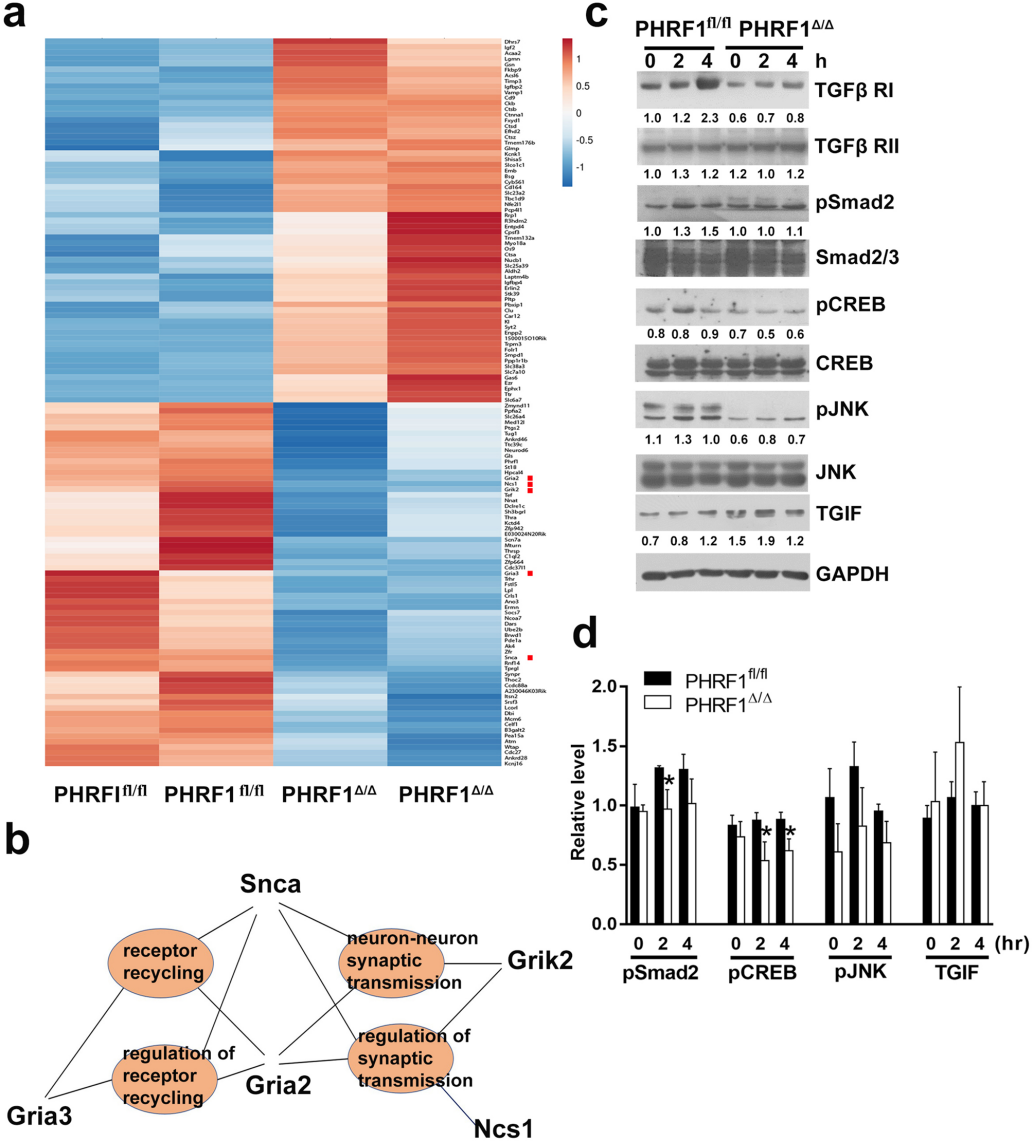
The altered expression of hippocampal genes involved in JNK and CREB signaling in PHRF1^Δ/Δ^ mice. (**a**) The Heatmap was generated using ClustVis by comparing top 65 up- and down-regulated genes in the hippocampi of 3-month-old PHRF1^fl/fl^ and PHRF1^Δ/Δ^ mice (n = 2 mice per group). Down-regulated genes are shown in blue shades and up-regulated genes are shown in red shades. (**b**) Identification of biological processes by Gene Ontology software based on the differentially down-expressed genes in PHRF1^Δ/Δ^ mice compared with PHRF1^fl/fl^ mice. (**c**) Hippocampal cells from 3-month-old PHRF1^fl/fl^ and PHRF1^Δ/Δ^ mice (*n* = 3) were independently incubated with TGF-β1 (10 ng/ml) at indicated times and cell lysates were immunoblotted with indicated antibodies. Representative band intensity of phosphoproteins was measured by ImageJ software and normalized with their corresponding internal controls. All Western blots were processed in identical conditions and cropped from Supplementary Fig. S4b. (**d**) Relative phosphorylation levels of pSmad2, pCREB, and pJNK and the amount of TGIF were measured and quantified with their internal controls. Data are mean ± SEM. Student t-test, *p < 0.05. n = 3.

We next evaluated the behavioral phenotypes of PHRF1^Δ/Δ^ mice in the aspect of learning and memory. A novel object recognition test was conducted after the open field test. The discrimination ratios between two identical objects were close to 1 in both PHRF1^fl/fl^ and PHRF1^Δ/Δ^ mice (Fig. 3a), indicating a minimal bias in the testing environment. However, when one object was replaced by a novel one, PHRF1^fl/fl^ mice spent more time exploring the novel object than the familiar one, rendering a high discrimination ratio. This ratio was much lower in PHRF1^Δ/Δ^ mice (Fig. 3a), indicating the impaired recognition memory in these mutants. A Y-maze test was then performed. PHRF1^Δ/Δ^ mice displayed a reduction of spontaneous alternations in the Y-maze test (Fig. 3b), indicating a faulty spatial working memory in these mice. We also conducted the Morris water maze to evaluate the capability of spatial learning and memory in PHRF1^Δ/Δ^ mice. At the beginning, PHRF1^fl/fl^ and PHRF1^Δ/Δ^ mice spent the same amount of time finding the underwater platform, indicating similar visual and mobile capabilities in both genotypes. However, as the training proceeded, from day 2 to day 5, different spatial learning patterns between two groups were noticed (Fig. 3c). PHRF1^Δ/Δ^ mice exhibited a longer latency to locate the underwater platform compared with PHRF1^fl/fl^ mice, suggesting a poor spatial learning capacity in these mutants. Subsequently, we tested the spatial memory after the 5-day training session. During this probe test, the underwater platform was removed. PHRF1^fl/fl^ mice swam directly to the area where the platform was used to be and searched for it, giving a number of entrances to this area; however, the number of entrances was much lower in PHRF1^Δ/Δ^ mice (Fig. 3d), indicating a defective spatial memory. The gender differences in PHRF1^Δ/Δ^ mice were not associated with impaired spatial memory, since male and female mice displayed similar patterns (Supplementary Fig. S2). Together, these tests showed behavioral phenotypes of PHRF1^Δ/Δ^ mice including elevated anxiety levels and impaired learning and memory. The behavioral features in PHRF1^Δ/Δ^ mice seemed to be associated with an altered structure and function of the hippocampus**.** We then explored the characteristics of hippocampal CA1 pyramidal neurons in anatomical (Fig. 4, Table 1), physiological (Fig. 5), and molecular (Fig. 6) aspects.

**Table 1:** Morphological parameters of dendritic structures. Morphometric features were compared between genotypes. n ≥ 30 neurons from 7 mice of each genotype. Data are mean ± SEM. **p < 0.01; ***p < 0.001. Two-tailed unpaired Student’s t-test.

	Apical dendrites		Basilar dendrites	
	PHRF1^fl/fl^	PHRF1^Δ/Δ^	PHRF1^fl/fl^	PHRF1^Δ/Δ^
Bifurcation nodes	23.34 ± 9.24	16.06 ± 0.79***	17.97 ± 1.16	11 ± 0.64***
Terminal ends	24.29 ± 1.50	16.88 ± 0.83***	20.87 ± 1.16	13.61 ± 0.63***
Highest order	13.24 ± 0.52	11.27 ± 0.41**	6.74 ± 0.28	5.7 ± 0.24**
Dendritic length (μm)	1599.76 ± 75.41	1015.19 ± 50.36***	1144.55 ± 48.61	686.62 ± 32.80***

### Dendritic structures of CA1 pyramidal neurons are altered in PHRF1^Δ/Δ^ mice

To address the consequences of PHRF1 loss in the hippocampus, the dendritic features of CA1 pyramidal neurons were analyzed (Fig. 4a). The complexity and length of dendritic arbors in CA1 pyramidal neurons were affected by the removal of PHRF1. The numbers of bifurcation nodes and terminal endings, as well as the highest order, were significantly reduced in both apical and basilar dendrites of CA1 pyramidal neurons collected from PHRF1^Δ/Δ^ mice (Table 1). For Sholl analysis, the numbers of intersections between dendritic tree and concentric rings were quantified at different distance from the soma. In both apical and basilar dendrites, the number of intersections, an index of dendritic complexity, was largely reduced in PHRF1^Δ/Δ^ group (Fig. 4b). A similar result was obtained by counting the numbers of dendritic segments at different orders (Fig. 4c). We further measured the lengths of dendritic arbors. The total dendritic length was largely decreased in relation to the ablation of PHRF1 (Table 1). This might be attributed to the decrease of dendritic branches (Fig. 4c) and reduction of internodal segment length (Fig. 4d). These results indicated that the elongation and bifurcation of dendrites are affected in the absence of PHRF1, resulting in shorter and less complicated dendrites in the CA1 pyramidal neurons of PHRF1^Δ/Δ^ mice. Furthermore, we calculated the density of dendritic spines on the dendrites. Interestingly, similar spine densities in both apical and basilar dendrites on CA1 neurons were found in PHRF1^fl/fl^ and PHRF1^Δ/Δ^ mice (Fig. 4e). Collectively, the morphological alterations of hippocampal neurons in PHRF1^Δ/Δ^ mice exhibited significant changes of hippocampal dendrites, possibly thereby reducing the dendritic surface area and number of dendritic spines in receiving external inputs.

### TGF-β1 failed to prolong synaptic potentiation in PHRF1^Δ/Δ^ mice

To clarify the electrophysiological properties of CA1 neurons in PHRF1^Δ/Δ^ mice, hippocampal slices were prepared to examine the synaptic transmission of CA1 pyramidal neurons. The field excitatory postsynaptic potential (fEPSP) was recorded from CA1 slices taken from control and PHRF1^Δ/Δ^ mice. The result showed that the fEPSP of PHRF1^Δ/Δ^ mice is not significantly different from that of PHRF1^fl/fl^ mice as the stimulus intensity increased (Supplementary Fig. S3a). Next, we measured the responses to paired-pulse facilitation (PPF), a form of short-term synaptic plasticity at the Schaffer collateral-CA1 synapse^15^. Again, no difference in PPF between the PHRF1^fl/fl^ and PHRF1^Δ/Δ^ mice was noticed (Supplementary Fig. S3b), suggesting that PHRF1 ablation does not affect short-term presynaptic plasticity. We compared the properties of long-term potentiation (LTP) of fEPSP in hippocampal CA1. The application of high frequency-stimulation immediately induced a post-tetanus potentiation (PTP), followed by a LTP of fEPSP in slices. However, no difference in LTP was noticed between the PHRF1^fl/fl^ and PHRF1^Δ/Δ^ mice (Supplementary Fig. S3c).

Since PHRF1 is involved in TGF-β signalling^10^ and the addition of TGF-β1 is able to prolong the synaptic potentiation induced by weak stimuli in the CA1^16^, we speculated whether synaptic plasticity was affected by PHRF1 in the presence of TGF-β. To test this, a weak theta burst stimulation (TBS) was used. Unlike high frequency-stimulation, weak TBS failed to induce LTP in both PHRF1^fl/fl^ and PHRF1^Δ/Δ^ mice. TBS only induced PTP and the fEPSP gradually declined to the baseline level (Fig. 5a). Notably, after the incubation of TGF-β1 (10 ng/ml) for 15 min, the fEPSP was potentiated for at least 60 min following the same TBS protocol in PHRF1^fl/fl^ mice but not in PHRF1^Δ/Δ^ mice (Fig. 5b). TBS-induced synaptic change in PHRF1^Δ/Δ^ mice quickly declined to the basal level (Fig. 5b,c), indicating that the down-regulated TGF-β signaling may contribute to a defect in synaptic plasticity in the hippocampal neurons of PHRF1^Δ/Δ ^mice.

### Ablation of PHRF1 induces changes in gene expression

To gain more insight into the molecular mechanism by which PHRF1 regulates characteristics of pyramidal neurons, we isolated hippocampal mRNAs to measure global gene expression in PHRF1^fl/fl^ and PHRF1^Δ/Δ^ mice using RNA-seq analysis. Approximately 450 differentially expressed genes were obtained and the heatmap was generated by analyzing top 65 up- and down-regulated genes based on log2 fold change, in which PPEE < 0.05 is considered as statistically significant (Fig. 6a). Top 65 down-regulated genes were further clustered for gene ontology analysis and a number of genes could be grouped into several biological processes (Supplementary Table S1). Among these, functional enrichments were visualized by Cnetplot. Interestingly, several distinctive biological processes featured in Gene Oncology (GO), such as regulation of synaptic transmission (GO#0051966, P = 0.0008), modulation of synaptic transmission (GO#0050804, P = 0.0008), receptor recycling (GO#0001881, P = 0.0002), and neuron-neuron synaptic transmission (GO#0007270, P = 0.0001) contain down-regulated genes Grik2, Gria2, Gria3, Ncs1, and Snca (indicated with red squares in Fig. 6a,b). To correlate the molecular basis on TGF-β1-mediated synaptic potentiation, we examined the phosphorylation levels of phospho-SMAD2 at S465/S467 (pSMAD2) in TGFβ canonical pathway and phospho-JNK at T183/T185 (pJNK) in the TGF-β non-canonical pathway. Phospho-CREB at S133 (pCREB), which is implicated in regulating various forms of synaptic plasticity and memory^17^, was also examined. Immunoblot analysis showed that the normalized phosphorylation level of pSMAD2 and pCREB were significantly decreased in PHRF1^Δ/Δ^ mice treated with TGF-β1. Although pJNK was reduced post TGF-β1 treatment in PHRF1^Δ/Δ^ mice, its P value was not statistically significant (Fig. 6c, dn = 3). Collectively, the down-regulated TGF-β signaling may contribute to the alterations of synaptic plasticity in the hippocampal CA1 neurons of PHRF1^Δ/Δ ^mice.

## Discussion

The dendritic architecture is essential for receiving external stimuli and formation of memory. Thus, dendritic abnormalities in neurons have the highest anatomical correlation with mental retardation and aging diseases. Genetic disorders such as Down, Williams, and Rubinstein-Taybi syndromes exhibit dendritic branching/spine abnormalities^18^. A decrease in the postsynaptic surface could result in the reduction of dendritic arborization and lead to neurologic impairments^19^. In this study, we describe an important link between PHRF1 and dendritic architecture of pyramidal neurons. The consequences of PHRF1 ablation in hippocampal CA1 neurons result in impaired dendritic complexity, reduced spatial memory, and increased anxiety-like behaviors in PHRF1^Δ/Δ^ mice.

TGF-β is a well-known neuroprotective and neurotrophic factor, and it also governs a wide variety of neurogenesis^20,21^. TGF-β promotes axon specification in the brain during development^22^ and guides the sprouting and elongation of neurites in the cell culture^23^. In TGF-β canonical signaling, TGF-β binding to TGFβRII induces the assembly of type I and type II receptors with the subsequent transphosphorylation of the type I receptor by the type II receptor kinase. The subsequent activation of the type I receptor leads to phosphorylation of SMAD2/SMAD3 proteins accompanied with SMAD4, that translocate into the nucleus to regulate the expression of different target genes involved in cell proliferation and neuronal survival^24,25^. The administration of TGF-β1 is able to prolong the effect of weak electrical stimuli on the synaptic potentiation in pyramidal CA1 neurons^16^. Interestingly, a similar result was observed in our control, but not in PHRF1^Δ/Δ^ mice. Our result revealed that PHRF1 is required in TGF-β1-mediated, long-term synaptic plasticity in hippocampal CA1 neurons. Consistently, PHRF1 ablation results in defective spatial memory and increased anxiety related behaviors in PHRF1 mutant mice. However, the absence of PHRF1 had little effect on short-term presynaptic plasticity or TBS-induced PTP. There are three distinct domains in PHRF1: an N-terminal RING E3 ligase domain that ubiquitinates substrates, a PHD domain that recognizes methylated histones, and a C-terminal SRI domain to interact with Rpb1 of RNA Pol II complex. Further studies to determine which domains are required would shed light on elucidating the role of PHRF1 in dendritic arborization and synaptic plasticity.

In support of a connection between PHRF1 depletion and synaptic plasticity, we observed a substantial decrement in some genes involved in the regulation of synaptic transmission according to the biological process of Gene Ontology analysis (Fig. 6b). It is intriguing to note that NGS analysis identified several gene products with established functions in the regulation of synaptic transmission. Among these differentially-expressed genes, the mRNA levels of the glutamate ionotropic receptor AMPA type subunit 2 (Gria2), Gria3, and the glutamate ionotropic receptor Kainate type subunit 2 (Grik2) were reduced in the hippocampi of PHRF1^Δ/Δ^ mice. Glutamate receptors are the predominant excitatory neurotransmitter receptors in the mammalian brain and are activated in a variety of learning and memory processes. Its downregulation leads to defective ion influx^26^, which might be associated with the defects in learning and memory and in concert with our results in PHRF1^Δ/Δ^ mice.

Another interesting finding is the decreased phosphorylation of pCREB and pJNK, which have been shown to play important roles in LTP and spatial memory^24,25^. In the non-canonical TGF-β signaling pathway, TGF-β receptors activate JNK and p38 MAPK specifically by MAP kinase kinases (MKKs) MKK4 or MKK3/6, respectively^27–29^. By contrast, the phosphorylation of CREB at S133 is predominantly controlled by protein kinase A (PKA) or Ca^2+^/calmodulin-dependent kinase (CaMK). CREB interacts with a CREB-binding protein (CBP), a transcriptional co-activator, only when S133 is phosphorylated, thereby inducing the gene transcription required for LTP and long-term memory^30–32^. TGF-β1 has been identified to induce LTP and increase CREB phosphorylation at S133 in CA1 neurons; SB431542, a specific TGF-β1 inhibitor could reduce the pCREB level, impairs LTP and memory^16^. Furthermore, TGF-β also induces a robust CREB phosphorylation at S133 in mouse embryonic fibroblasts and this phosphorylation is inhibited by VX745, a selective and potent inhibitor of p38 MAPK. Therefore, TGFβ-induced activation of p38 MAPK is necessary and sufficient for the activation of pCREB^33^. Since both pCREB and pJNK were decreased in PHRF1^Δ/Δ^ CA1 extracts (Fig. 6c), it is highly plausible to conclude that PHRF1 deficiency mainly results in a lower non-canonical TGF-β signaling pathway to reduce the phosphorylation of pJNK and pCREB.

Finally, the role TGF-β signaling in neurological disorders has been discussed^34^. Among various mental disorders related to TGF-β signaling, reduced serum TGF-β level is associated with major depression^35^. It is in line with our finding that in PHRF1^Δ/Δ^ mice, anxiety-like behaviors were noted, while TGF-β signaling was downregulated. Thus, the neuroprotective features of TGF-β1 would be dampened by the loss of PHRF1. In summary, our study demonstrated for the first time, that PHRF1 has a substantial impact on the dendritic architecture, synaptic plasticity, memory and mood regulation.

## Materials and methods

### PHRF1 conditional knockout mice

All animal studies were performed in compliance with the guidelines of the Institutional Animal Care and Use Committee, Academia Sinica. Two loxp elements flanking exon 2 and 9 (a.a. 1–343) were introduced into a murine *PHRF1* gene by the Crispr/Cas9 method. Floxed homozygous male and female mice (PHRF1^fl/fl^) were crossed with Camk2a-iCre transgenic mice to produce PHRF1^Δ/Δ^ mice. In the current study, 8–10 weeks old young adult mice of both sex were used.

### Genotyping

DNA samples were extracted from mouse tails using the EasyPure Genomic DNA spin kit (Bioman, Taipei, Taiwan). The following primers were synthesized for genotyping; 5VF2, 5′-TTGCAAAGGAGGACTAGCAGGT-3′. 5VR1, 5′-CTTCGTGCCTGAGGACTAGC-3′. Camk2aF1, 5′-GGTTCTCCGTTTGCACTCAG-3′. iCreR1, 5′-TCCCTCACATCCTCAGGTTC-3′. 5VF2 and 5VR1 primers were used to amplify the control and floxed PHRF1 alleles. Camk2aF1 and iCreR1 primers were used to identify the presence of Camk2a-iCre.

### Behavioral tests

Prior to the start of each behavioral test, the mice were brought to the test place for habituation for at least 30 min. More than five mice of each group were used. All experiments were performed in agreement with a protocol approved by the Institutional Animal Care and Use Committees at Academia Sinica.

#### Open-field test^36^

An individual mouse was placed in a novel open-field arena (40 × 40 cm) and allowed to explore freely for 30 min. Their behavior was continuously recorded by a video camera placed above the arena. The distances travelled and time spent within the central and peripheral areas were analyzed by TopScan LITE software (Clever Systems, Reston, VA, USA).

#### Elevated-plus maze (EPM) test

The EPM test was used to evaluate anxiety-like behavior in mice^37^. During the test, a mouse was placed on the central platform of the maze with its head facing one of the open arms and allowed to move freely for 10 min. The behavior was recorded by a video camera and analyzed with TopScan LITE (Clever Systems). The distance traveled and time spent in the open arms, closed arms, and central region were quantified.

#### Light/dark box test

Anxiety-related behavior was assayed using the light/dark box test^38^. The averseness of the light compartment was enhanced by increasing additional illumination up to 400 lx above the center of the light area. Each mouse was placed in the light area, facing away from the opening and allowed to explore the box for 5 min. Dependent variables included the time spent in light area, the first entrance latency to dark area (all four paws in), and the total number of transitions between the two areas.

#### Y-maze test

Short-term spatial working memory in mice was assessed by counting the spontaneous alternations in a Y-maze, which was conducted as described previously. Briefly, a mouse was placed at the end of one arm of the Y-maze and allowed to move within three arms freely during an eight-minute period. The total number and series of arm entries were recorded. The number of non-overlapping entrance sequences (e.g,. ABC, BCA) was defined as the number of alternations. Spontaneous alternation (%) was calculated as: (number of alternations)/(total number of arm entries-2) × 100.

#### Morris water maze

The properties of hippocampus-mediated spatial learning and memory were evaluated using the Morris water maze test, which was conducted as described previously^39^. Briefly, a circular pool contained water (19 °C) that was made opaque with non-fat milk. A hidden platform was placed at one cm below the water surface. During the training period, the mouse was allowed to swim for 90 s to locate the hidden platform. An additional 30-s was given so that the mouse could stand on the platform. Four trials at different start points (north, south, east, and west) were given each day for five consecutive days. After five-day training period, another probe test was given in which the platform was removed. Latencies to reach the hidden platform and the swim paths were recorded and analyzed.

#### Novel object recognition test

Short-term recognition memory was tested using a novel object recognition test^40^. Briefly, the mice were placed in an open-field arena for 30 min for habituation before the test day. During the exploration phase, a mouse was placed in the open field and presented with a pair of identical objects for 8 min. The mouse was then returned to its home cage for 10 min as a retention period. In the test phase, the mouse was returned to the arena and presented with one familiar object and one novel object. The time spent in exploration behavior was quantified. The discrimination ratio, (novel object exploration time/familiar object exploration time), was used to evaluate the performance of short-term recognition memory.

### Golgi-Cox impregnation and morphometric analyses

Golgi-Cox impregnation was conducted as previously described^41^. Briefly, after transcardiac perfusion with phosphate-buffered saline and fixative (4% paraformaldehyde in phosphate buffer, pH 7.4), whole brains were immersed in the impregnation solution from the FD Rapid Golgi Stain kit (NeuroTechnologies, Ellicott City, MD, USA). After three weeks of impregnation, brain samples were sectioned and incubated with a mixture of developer and fixer solutions (FD Rapid Golgi Stain kit). The pyramidal neurons in the hippocampal CA1 region were examined under a light microscope with a 20 × objective lens for dendritic morphology and 100 × objective lens for spine analysis. Series of pictures were taken by a CCD camera using the Stereo Investigator system (MBF Bioscience, Williston, VT, USA). The morphology of selected neurons was reconstructed and analyzed with Neurolucida software (MBF Bioscience). Data were expressed as the mean ± SEM. Two-tailed unpaired Student’s t-test was used for statistical analysis.

### Hippocampal slices and electrophysiology

Hippocampal slices and electrophysiology recordings were conducted as previously described^35^. Briefly, male young adult (8–10 weeks old) PHRF1^fl/fl^ and PHRF1^Δ/Δ^ mice were anesthetized with isoflurane and the brains were quickly placed in ice-cold artificial cerebrospinal fluid (ACSF). Hippocampal slices of 450 μm thickness were transversely cut and transferred to a holding chamber for recovery (> 90 min) before electro-recording. Slices were maintained at room temperature and oxygenated ACSF (5% CO_2, _95% O_2_) containing 0.1 mM picrotoxin (GABA_A_ receptor antagonist). The border between the CA1 and CA3 areas was cut to prevent an epileptiform discharge of pyramidal neurons. Field excitatory postsynaptic potentials (fEPSPs) were obtained by stimulating Schaffer collateral fibers with a bipolar electrodes (Frederick Haer Company, Bowdoinham, ME, USA) (10 μM impedance), and a recording in the CA1 stratum radiatum with a borosilicate glass electrode. Stable baseline fEPSP activity was recorded by applying a constant current pulse of 40 μs duration (DS3, Digitimer, Welwyn Garden City, UK) every 15 s for at least 15 min. A high frequency-stimulation protocol consisting of 3 trains of 100 pulses at 100 Hz with 10 s inter-train interval, was used to induce long-term potentiation (LTP). A weak theta burst stimulation (TBS) consisting of a 10-burst train of 4 pulses at 100 Hz with the bursts repeated at 5 Hz, was performed to test the effect of TGF-b. Synaptic responses were recorded for two hours after TBS and the initial slopes of the fEPSPs were measured and normalized to the average value of the baseline for data analysis. All signals were filtered at 2 kHz by a low-pass Bessel filter provided by the amplifier (Multiclamp 700 B; Axon Instruments, Union City, CA) and digitized at 5 kHz using CED micro 1,401 interface running Signal software provided by CED (Cambridge Electronic Design, Cambridge, UK). All data are presented as the mean ± SEM and were statistically compared using one-way ANOVA. *p < 0.05.

### Next generation sequencing

Male hippocampi were dissected and total RNA was extracted using Trizol reagent (Invitrogen). mRNAs were purified using poly-T oligo-attached beads and fragmented for cDNA synthesis. cDNAs were sequentially synthesized and a single ‘A’ nucleotide is added to 3′ end of cDNAs. Multiple indexing adapters are ligated to 5′ and 3′ of the ends of the cDNAs. A library was constructed and validated on the Agilent 2,100 Bio-analyzer and Real-Time PCR System. Subsequently, Illumina NovaSeq sequencing was proceeded. The RNA-seq reads were trimmed to remove the adaptor and low-quality sequences by using Trimmomatic. The trimmed reads were mapped to the mouse reference genome sequence (mm10) using Bowtie2. Quantification of gene expression was performed by EBseq. The Heatmap was generated by online ClustVis (https://biit.cs.ut.ee/clustvis/). Gene set enrichment analysis was performed to provide more information about the biological functions and pathways significantly enriched in up- or down-regulated genes (DEGs) by focusing on gene ontology (GO) term (BP, biological process) and Kyoto Encyclopedia of Genes and Genomes (KEGG) pathways using Cluster Profiler.

### Immunoblotting

Hippocampi dissected from hemi-brains of mice were homogenized and lysed. After centrifugation at 13,000 rpm, protein concentrations were measured using the BCA protein assay kit (Pierce). Lysates were separated on a 4–12% Bis–Tris gels, blotted onto membranes, and analyzed with indicated antibodies.

### Antibodies

Rabbit anti-mPHRF1 polyclonal antibody was raised against 6xHis tagged recombinant PHRF1protein (a.a. 1,450–1,682). Mouse anti-α-tubulin was purchased from Santa Cruz Biotech (Santa Cruz, CA). Rabbit anti-phospho Smad2/CREB/JNK antibodies were from Cell Signaling (Danvers, MA). Rabbit anti-TGFbRI/TGFbRII antibodies were obtained from Abcam (Cambridge, MA). Rabbit anti-SMAD2/3 antibody was from GeneTex (Hsinchu, Taiwan). Mouse anti-GAPDH antibody was from Novus (Littleton, CO).

## Supplementary information

Supplementary file1 (DOCX 847 kb)
