# Supplementary material for: An important role of PHRF1 in dendritic architecture and memory formation by modulating TGF-β signaling
Source: Sci Rep. 2020 Jul 2;10:10857. doi: 10.1038/s41598-020-67675-2 (PMC7331665; doi:10.1038/s41598-020-67675-2)
Supplement: Supplementary file 1 — Supplementary file1 (DOCX 847 kb) [file 41598_2020_67675_MOESM1_ESM.docx]

**Supplementary Information**

**Important roles of PHRF1 in dendritic architecture and memory formation**

Ting-Wei Shih^1,*^, Li-Jen Lee^2,3,4,*^, Ho-Ching Chang^2^, Hung-Wei Lin^1^, and Mau-Sun Chang^1,9,**^

**
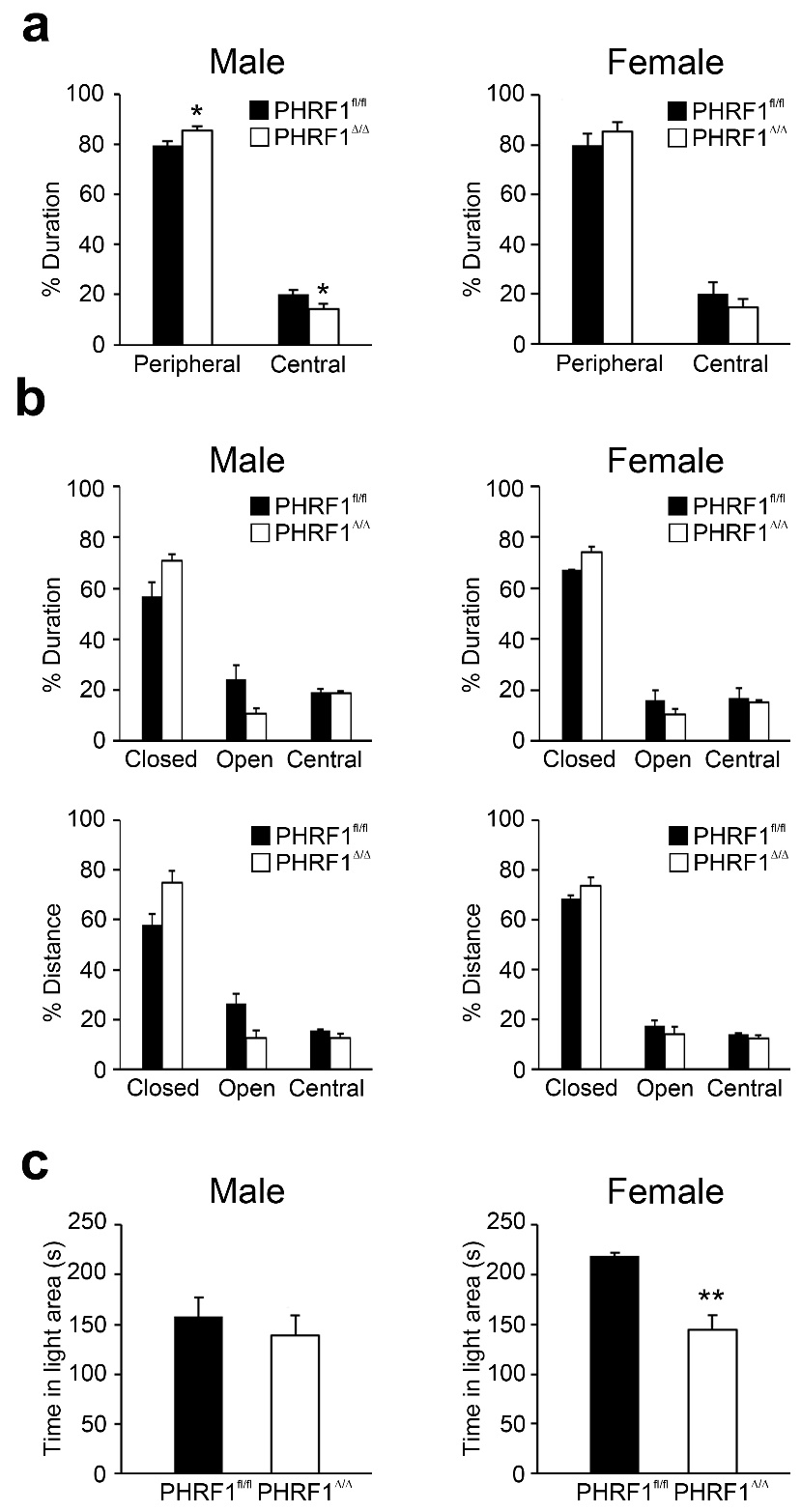
**

**Supplementary Figure S1. Male and female behavioral tests.** (a) Open field test. The duration spent in central and peripheral regions were quantified. (PHRF1^fl/fl^ n = 15, 12 males, 3 females; PHRF1^Δ/Δ^ n = 13, 10 males, 3 females) (b) Elevated-plus maze test. The percentage of time and distance spent in closed arm, open arm and central zones were analyzed. (PHRF1^fl/fl^ n = 6, 4 males, 2 females; PHRF1^Δ/Δ^ n = 7, 3 males, 4 females) (c) Light/dark exploration. The time spent in the light region between the two compartments was quantified. (PHRF1^fl/fl^ n = 7, 4 males, 3 females; PHRF1^Δ/Δ^ n = 7, 3 males, 4 females). Data are mean ± SEM. *p<0.05, **p<0.01, two-tailed unpaired Student t-test.

**
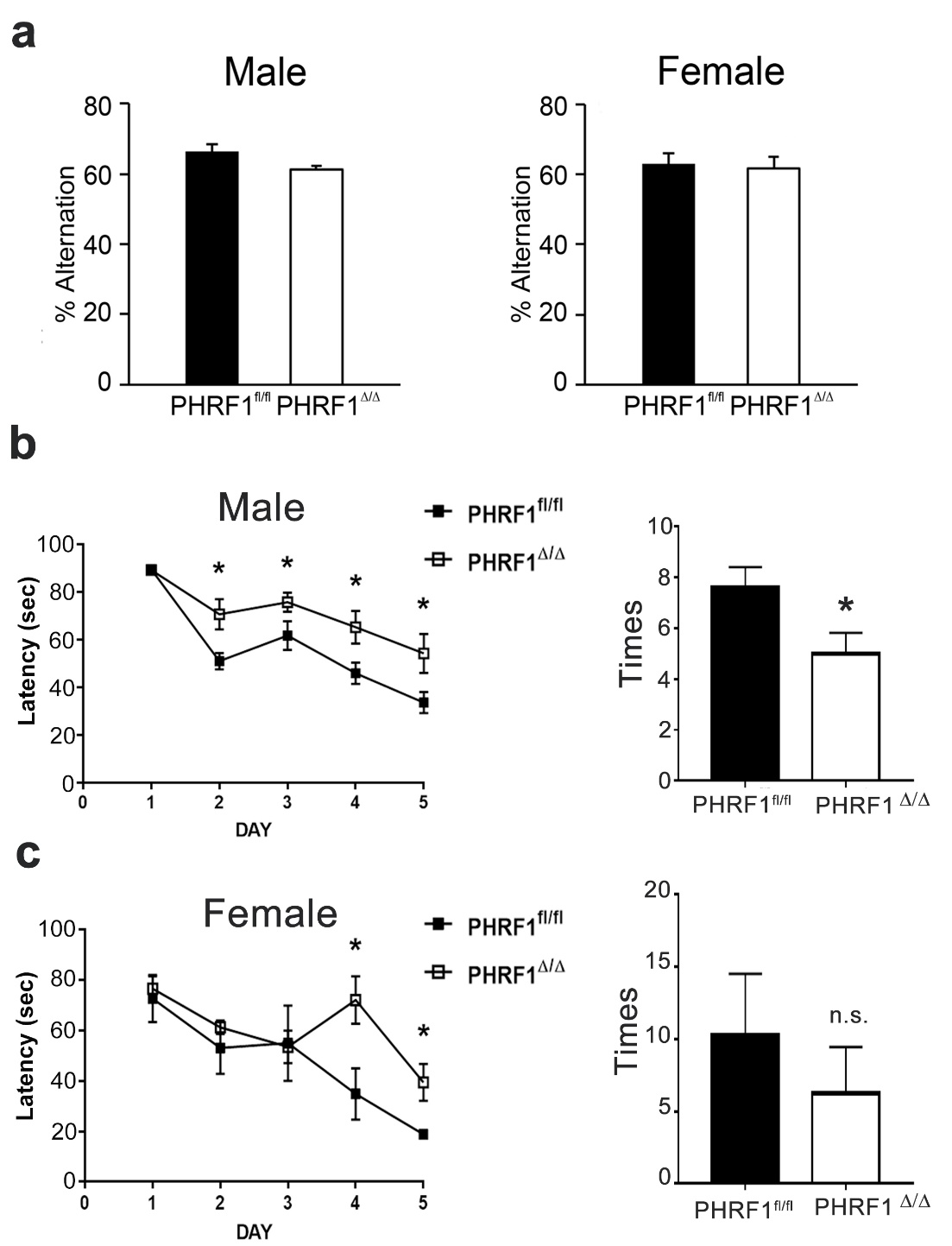
**

**Supplementary Figure S2.** Spatial memory in male and female mice. (a) Y-maze test. The number of alternations were analyzed. Data are mean ± SEM. *p < 0.05, two-tailed unpaired Student t-test. (PHRF1^fl/fl^ n = 13, 11 males, 2 females; PHRF1^Δ/Δ^ n = 12, 9 males, 3 females) (b) PHRF1^fl/fl^ and PHRF1^Δ/Δ^ male mice were placed in Morris water maze and the time to the hidden platform was quantified. Right panel, the probe trial on Day 6. * *p* < 0.05 by student’s t-test. n=8. (c) The time of PHRF1^fl/fl^ and PHRF1^Δ/Δ^ female mice was also quantified. Right panel, the probe trial on Day 6. * *p* < 0.05 by student’s t-test. n=4. n.s., not significant.

**
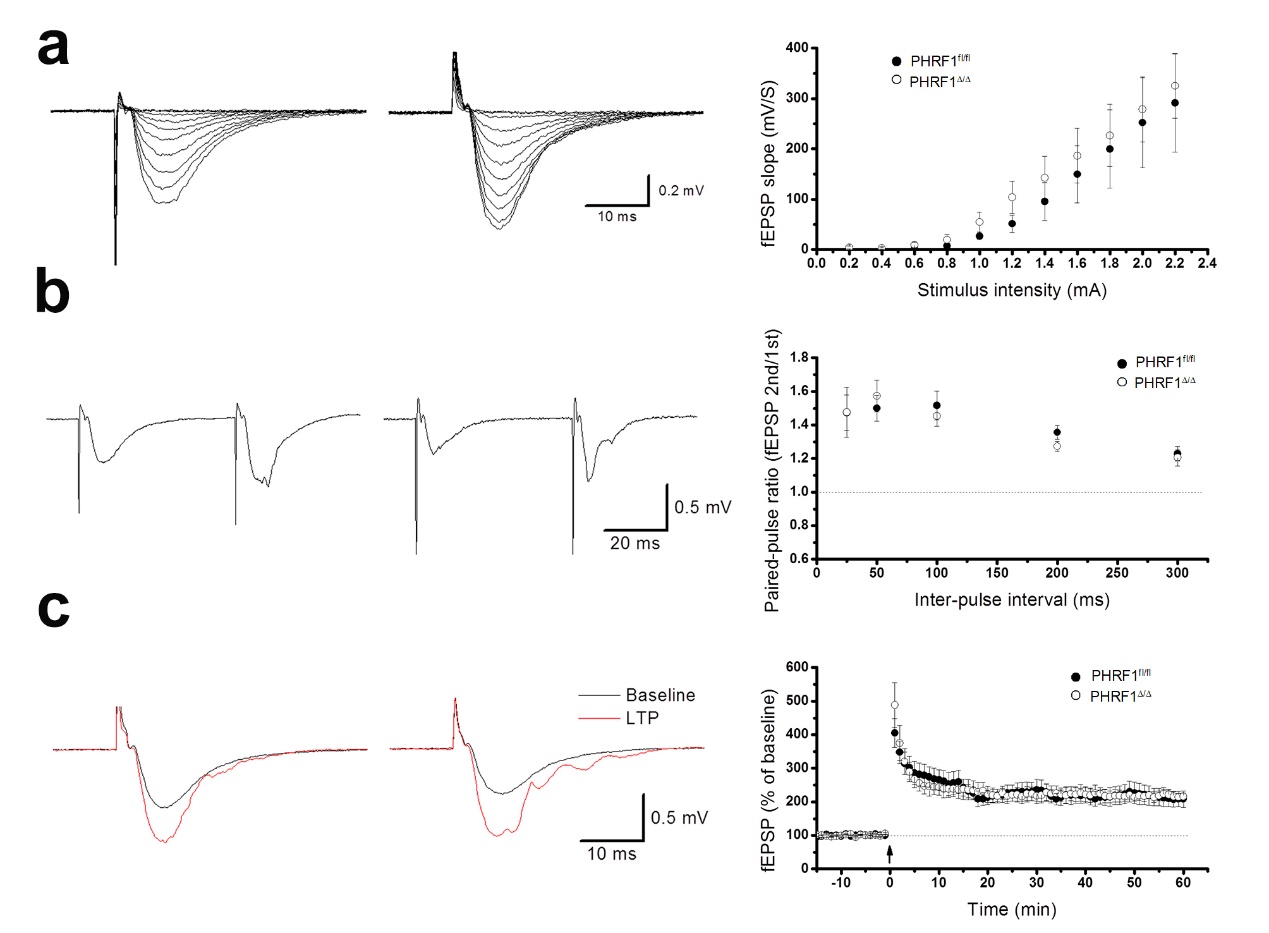
**

**Supplementary Figure S3.** The field EPSP (fEPSP) recording at CA1 synapses in hippocampal slices. (a) Basal synaptic transmission was conducted and stimulus intensity was plotted against the slopes of fEPSP for 8-week-old mice (n=7 slices from 3 PHRF1^fl/fl^ male mice; n=7 slices from 3 male PHRF1^Δ/Δ^ mice). (b) Paired pulse facilitation. Four inter-stimulus intervals were recorded to show the percentage of paired pulse facilitation (n = 7 slices from 3 PHRF1^fl/fl^ male mice; n = 7 slices from 3 male PHRF1^Δ/Δ^ mice). (c) LTP. An arrow indicates the start of high-frequency stimulation (3 trains of 100 pulses at 100 Hz with inter-train interval being 10 s). Note that there is no statistical difference in these experiments. (n = 7 slices from 3 male PHRF1^fl/fl^ mice; n = 7 slices from 3 male PHRF1^Δ/Δ^ mice)

**Supplementary Table S1.** List of representative differentially down-regulated genes using GOterm analysis in PHRF1^Δ/Δ^ mice compared with PHRF1^fl/fl^ mice.

| **GO.Term** | **Description** | **count** | | **GeneRatio** | **P value** | **P.adjust** | **Gene** |
| --- | --- | --- | --- | --- | --- | --- | --- |
| GO:0051966 | regulation of synaptic transmission, glutamatergic | | 3 | 3/63 | 0.000817313 | 0.096917387 | Gria2//Grik2//Ptgs2 |
| GO:0050804 | modulation of synaptic transmission | | 6 | 6/63 | 0.000837563 | 0.096917387 | Snca//Gria2//Dbi//Ncs1  //Grik2//Ptgs2 |
| GO:0001881 | receptor recycling | | 3 | 3/63 | 0.000157538 | 0.052735866 | Snca//Gria2//Gria3 |
| GO:0001919 | regulation of receptor recycling | | 3 | 3/63 | 6.42056E-05 | 0.031637816 | Snca//Gria2//Gria3 |
| GO:0050806 | positive regulation of synaptic transmission | | 6 | 6/63 | 3.13927E-05 | 0.031637816 | Snca//Gria2//Dbi//Ncs1  //Grik2//Ptgs2 |
| GO:0032225 | regulation of synaptic transmission, dopaminergic | | 2 | 2/63 | 0.000940946 | 0.096917387 | Snca//Ptgs2 |
| GO:0033151 | V(D)J recombination | | 2 | 2/63 | 0.000940946 | 0.096917387 | Atm//Dclre1c |
| GO:0034397 | telomere localization | | 2 | 2/63 | 0.000940946 | 0.096917387 | Ube2b//Atm |
| GO:0007270 | neuron-neuron synaptic transmission | | 5 | 5/63 | 7.08838E-05 | 0.031637816 | Snca//Gria2//Dbi//Grik2  //Ptgs2 |
| GO:0090220 | homologous chromosome segregation | | 2 | 2/63 | 0.000831684 | 0.096917387 | Ube2b//Atm |
| GO:0045141 | meiotic telomere clustering | | 2 | 2/63 | 0.000728985 | 0.096917387 | Ube2b//Atm |
| GO:0033127 | regulation of histone phosphorylation | | 2 | 2/63 | 0.000632882 | 0.096917387 | Ube2b//Atm |
| GO:0006638 | neutral lipid metabolic process | | 4 | 4/63 | 0.000252802 | 0.067700469 | Snca//Dbi//Thrsp//Lpl |

**
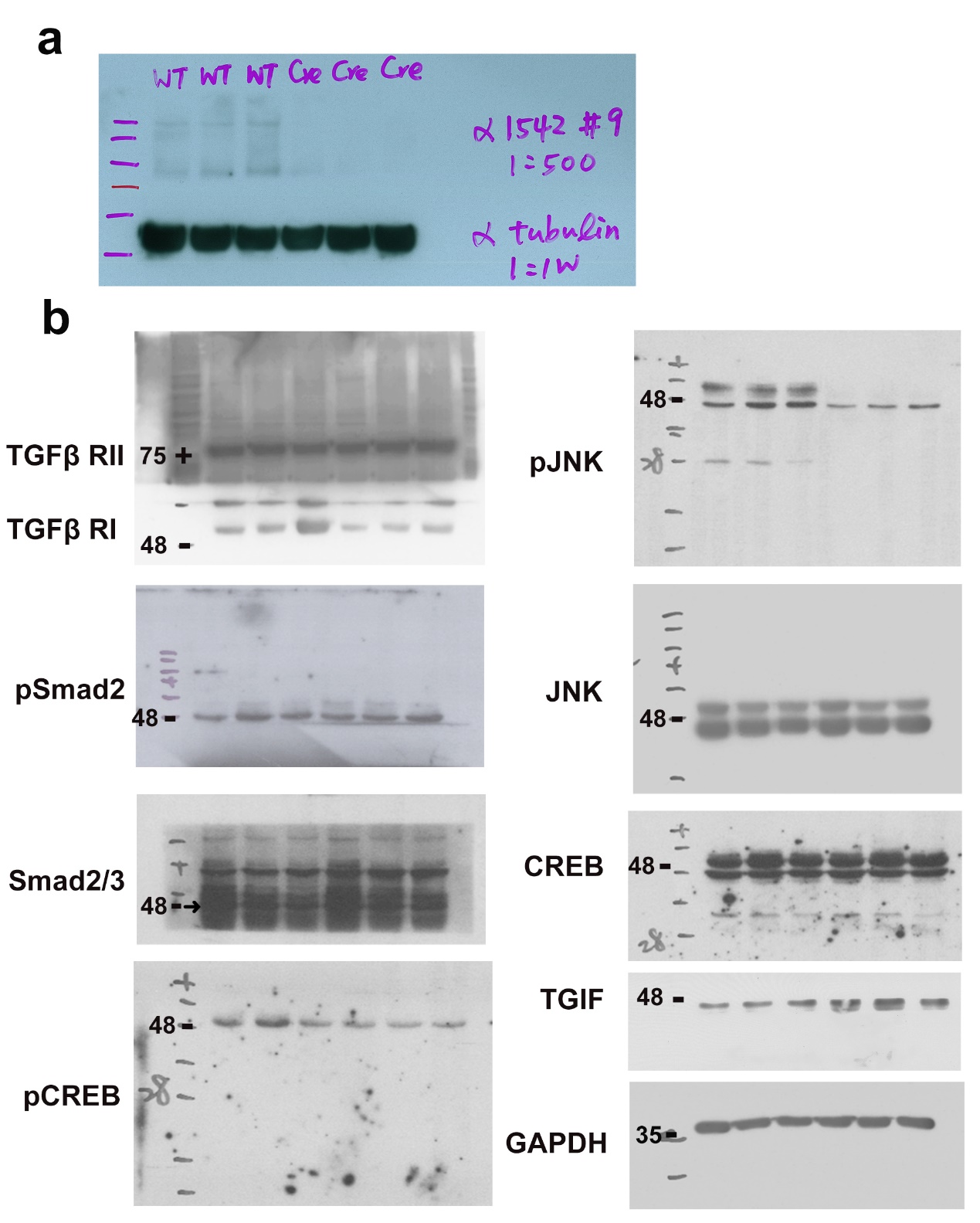
**

**Supplementary Figure S4.** Uncropped images for all gels and Western blots.
